# Möglichkeiten der funktionellen Bildgebung bei Tinnitus

**DOI:** 10.1007/s00106-023-01319-5

**Published:** 2023-06-29

**Authors:** Benjamin Isler, Patrick Neff, Tobias Kleinjung

**Affiliations:** 1 Klinik für Ohren‑, Nasen‑, Hals- und Gesichtschirurgie, Universitätsspital Zürich, Universität Zürich, Zürich, Schweiz; 2 Klinik für Psychiatrie und Psychotherapie, Universität Regensburg, Regensburg, Deutschland; 3 Neuro-X Institute, École Polytechnique Fédérale de Lausanne (EPFL), Geneva, Schweiz; 4 Centre for Cognitive Neuroscience, Universität Salzburg, Salzburg, Österreich; 5 Klinik für Ohren‑, Nasen‑, Hals- und Gesichtschirurgie, Universitätsspital Zürich, Frauenklinikstr. 24, 8091 Zürich, Schweiz

**Keywords:** Funktionelle Bildgebung des Gehirns, Diagnostische Bildgebung, Positronenemissionstomographie-Computertomographie, Elektroenzephalographie, Magnetresonanztomographie, Functional brain imaging, Diagnostic imaging, Positron emission tomography computed tomography, Electroencephalography, Magnetic resonance imaging

## Abstract

**Hintergrund:**

Die Pathophysiologie des Tinnitus ist nach wie vor nicht ausreichend verstanden. Verschiedene Bildgebungsmethoden helfen beim besseren Verständnis der komplexen Zusammenhänge, die zur Wahrnehmung von Tinnitus führen.

**Ziel der Arbeit:**

Es erfolgt die Vorstellung von verschiedenen funktionellen Bildgebungsmethoden, die in der Erforschung von Tinnitus eingesetzt werden können.

**Material und Methoden:**

Unter Einbezug der aktuellen Fachliteratur zum Thema gehen die Autoren auf die relevanten Bildgebungsmethoden der Tinnitusforschung ein.

**Ergebnisse und Schlussfolgerung:**

Die funktionelle Bildgebung kann Korrelate von Tinnitus aufzeigen. Aufgrund der noch eingeschränkten zeitlichen und räumlichen Auflösung der aktuellen Bildgebungsmodalitäten lässt eine abschließende Erklärung von Tinnitus auf sich warten. Mit der weiteren Verbreitung der funktionellen Bildgebung lassen sich in Zukunft zusätzliche wichtige Erkenntnisse zur Aufklärung von Tinnitus gewinnen.

## Abstract (en)

**Background:**

The pathophysiology behind tinnitus is still not well understood. Different imaging methods help in the understanding of the complex relationships that lead to the perception of tinnitus.

**Objective:**

Herein, different functional imaging methods that can be used in the study of tinnitus are presented.

**Materials and methods:**

Considering the recent literature on the subject, the relevant imaging methods used in tinnitus research are discussed.

**Results and conclusion:**

Functional imaging can reveal correlates of tinnitus. Due to the still limited temporal and spatial resolution of current imaging modalities, a conclusive explanation of tinnitus remains elusive. With increasing use of functional imaging, additional important insights into the explanation of tinnitus will be gained in the future.

Tinnitus stellt hohe Anforderungen an die Medizin und die Forschung auf gleiche Weise: Seine große Variabilität sowie die fehlende Möglichkeit zur Objektivierung dieser Wahrnehmung erschwert die Arbeit von Forschern und Ärzten. Die funktionelle Bildgebung gibt Einblicke in die Neuroanatomie und Neuropathophysiologie und ermöglicht wichtige Erkenntnisse zur Entstehung und Aufrechterhaltung der Empfindung.

## Tinnitus als individuelle Erkrankung

Die Pathophysiologie hinter Tinnitus, der Wahrnehmung von Ohrgeräuschen in Abwesenheit einer externen Geräuschquelle, ist nach wie vor nicht ausreichend verstanden. Zahlreiche Faktoren erschweren die Erforschung der Erkrankung. Tinnitus kann sich in verschiedenen Geräuscharten, seitengetrennt oder beidseitig und in verschiedenen Intensitäten äußern. Darüber hinaus charakterisiert sich der Tinnitus durch Leidensdruck und vorhandene Bewältigungsstrategien, was ihn zu einer sehr individuellen Erkrankung macht. Häufige Komorbiditäten wie Hyperakusis oder psychische Einflussfaktoren wie Depression oder Stress und die meist bereits vorbestehende Hörminderung erschweren die Entschlüsselung des Krankheitsbilds.

Tinnitus ist häufig mit einem Hörverlust verbunden

Aktuelle Erkenntnisse aus Studien am Menschen und aus der Tierforschung deuten darauf hin, dass die Pathophysiologie von Tinnitus komplizierter ist, als es die Intuition vermuten lässt. Tinnitus ist häufig mit einem Hörverlust verbunden, und die meisten pathophysiologischen Modelle gehen davon aus, dass Hörverlust der wichtigste Auslöser des Tinnitus ist. Die sich daran anschließenden Veränderungen im zentralen Nervensystem (ZNS) betreffen ein neuronales Netzwerk, das sowohl traditionell definierte auditorische Hirnareale als auch nichtauditorische Bereiche umfasst [18, 30]. Wichtige Erkenntnisse für das Verständnis der Tinnituspathophysiologie wurden am Tiermodell gewonnen, da hier invasive Methoden zum Einsatz kommen, die beim Menschen nicht angewendet werden können [4]. Beim Menschen hat der technologische Fortschritt in den strukturellen und funktionellen Neuroimagingverfahren wesentlich zum Verständnis der Tinnituspathophysiologie und den damit verbundenen Komorbiditäten beigetragen. Diese Methoden versprechen auch in Zukunft weitere Einblicke in die Pathophysiologie und in die Entstehung von Tinnitus und können in der Entwicklung von Therapien einen entscheidenden Faktor darstellen.

Unter dem Begriff der funktionellen Bildgebung in Bezug auf das Gehirn (deshalb syn. „Neuroimaging“) versteht man die Darstellung von Hirnaktivität und physiologischen Prozessen mit Bezug zu anatomischen Strukturen [17]. Somit eignet sich die funktionelle Bildgebung sehr gut zur Entdeckung komplexer neuronaler Mechanismen bei TinnituspatientInnen. Funktionelle Bildgebungsverfahren bieten eine Möglichkeit, Eigenschaften der neuronalen Aktivität wie Ort, Stärke und funktionelle Verbindungen zu beschreiben, die innerhalb des ZNS in einem zeitlichen Zusammenhang stehen. Im Gegensatz dazu dient die strukturelle Bildgebung der Darstellung von strukturell-anatomischen Unterschieden sowohl in den auditorischen als auch in den nichtauditorischen Regionen im Vergleich zwischen Tinnitusbetroffenen und ProbandInnen ohne Tinnitus. Insbesondere wurden in Studien mittels voxelbasierter oder oberflächenbasierter Morphometrie (beide gestützt auf strukturelle Magnetresonanztomographie[MRT]-Scans) Veränderungen in der grauen und weißen Substanz beobachtet, die sowohl als Erklärung für den mit Tinnitus assoziierten Hörverlust dienen könnten wie auch der Tinnituskomponente selbst geschuldet sein könnten [22, 28, 29].

Die Studienmodelle mit funktioneller Bildgebung ermöglichen den Forschern eine große Bandbreite an Untersuchungen und können mit aktivem Einbezug des Probanden oder auch völlig passiv erfolgen. Bei Tinnitusstudien ist in aller Regel keine Mitarbeit der ProbandInnen erforderlich, was durch Minimierung von weiteren Einflussfaktoren ein Vorteil gegenüber reinen Verhaltensstudien ist.

Bei den funktionellen Methoden unterscheidet man direkte Techniken wie die Elektroenzephalographie (EEG) und die Magnetenzephalographie (MEG), welche durch Neuronen ausgelöste Hirnströme ableiten, von den indirekten Verfahren. Hierzu zählen die funktionelle Magnetresonanztomographie (fMRT) und die Positronenemissionstomographie (PET). Diese messen über Veränderungen des Blutflusses oder der Energieaufnahme in bestimmten Bereichen des Gehirns die Aktivität von Neuronengruppen. In Tab. 1 sind diese Methoden zusammengefasst.

**Table 1:** 

Bildgebungsmethode	Messung der Gehirnaktivität	Auflösung zeitlich [17]	Auflösung räumlich [17]
fMRT	Indirekt	4–8 s	4–16 mm^3^
fMRS	Indirekt	Mehrere Minuten^a^	Gehirnregionen^a^
PET	Indirekt	Einige Minuten	2–8 mm^3^
EEG	Direkt	Millisekunden	10–20 mm^3^
MEG	Direkt	Millisekunden	10–20 mm^3^

*EEG *Elektroenzephalographie, *fMRT *funktionelle Magnetresonanztomographie, *fMRS *funktionelle Magnetresonanzspektroskopie, *MEG* Magnetenzephalographie, *PET* Positronenemissionstomographie

^a^Abhängig von dem zu untersuchenden Molekül

Die bildgebenden Verfahren werden durch aktive Neuromodulation wie transkranielle Magnetstimulation (TMS) erweitert. Mittels elektromagnetischer Induktion wird über einem bestimmten Hirnareal ein elektromagnetischer Impuls abgegeben. Damit kann die Aktivität des arbeitenden Gehirns in diesem Bereich (nichtinvasiv) beeinflusst werden.

Eine weitere Methode, welche zu den funktionell bildgebenden Verfahren gezählt wird, ist die Nahinfrarotspektroskopie. Diese misst lokale Blutflussveränderungen mittels Lichtstrahlen, die auf das Gehirn projiziert werden. Dabei passiert das Licht das Hirngewebe und kann mittels Sensoren auf der Kopfoberfläche gemessen werden. Dieses Verfahren erlaubt die Messung von Konzentrationen von oxygeniertem und desoxygeniertem Blut sowie dem zerebralen Blutfluss und gibt so indirekt Auskunft über aktive Hirnregionen. Aufgrund der Neuheit der Methode gibt es dazu noch nicht viele Untersuchungen bei Tinnitus [33].

Im vorliegenden Artikel wird ein Einblick in die Möglichkeiten der wichtigsten funktionellen Bildgebungsmethoden bei TinnituspatientInnen gegeben und aufgezeigt, welche relevanten Veränderungen im ZNS in Verbindung mit Tinnitus auftreten können. Darüber hinaus wird auch auf die Grenzen der jeweiligen Modalitäten hingewiesen.

## Funktionelle Bildgebungsmethoden

Die funktionelle Bildgebung dient der Darstellung von dynamischen Hirnprozessen. Eine gute Bildgebungsmethode verfügt dabei über eine ausreichende zeitliche sowie räumliche Auflösung, um aufeinanderfolgende Abläufe und die anatomische Beziehung nachvollziehen zu können.

Verschiedene Modalitäten werden für spezifische Fragestellungen eingesetzt

Da bisher keine Methode beide Anforderungen gleichzeitig mit zufriedenstellender Präzision erfüllt, werden verschiedene Modalitäten für spezifische Fragestellungen eingesetzt. Am häufigsten wird dabei in der Tinnitusforschung auf die fMRT, PET, EEG oder MEG zurückgegriffen [1]. Im Folgenden wird aufgezeigt, welche Veränderungen in der funktionellen Bildgebung bei Tinnitusbetroffenen gefunden werden können. Sämtliche der im Folgenden beschriebenen Erkenntnisse werden in aller Regel im Rahmen von wissenschaftlichen Studien gewonnen. Hierbei wird auf Gruppenniveau eine Gruppe von Tinnitusbetroffenen mit einer Gruppe von ProbandInnen ohne Tinnitus verglichen.

### Funktionelle MRT

**Figure 1:**
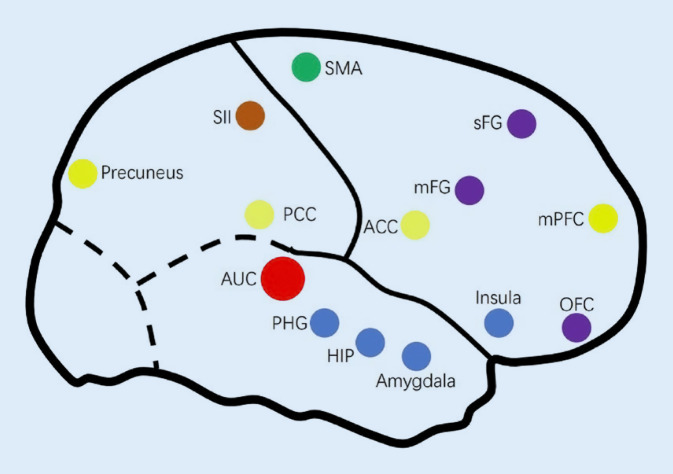

Mittels einer BOLD(Blood-Oxygenation-Level-Dependent)-Sequenz lässt sich das Verhältnis zwischen oxygeniertem und desoxygeniertem Blut nachweisen. Dies lässt Rückschlüsse auf Bereiche im Gehirn mit hoher Aktivität zu. Die Auflösung ist dabei sowohl zeitlich auf einige Sekunden als auch räumlich auf einige Millimeter begrenzt und sollte als Aktivität für bestimmte Regionen interpretiert werden [7]. Die Hirnaktivität kann in Ruhe („Resting-State-fMRT“) oder während der Erledigung einer Aufgabe („Task-Based-fMRT“), im Fall von Tinnitus am ehesten im Sinne einer auditiven Aufgabe, erfasst werden. Bei der Resting-State-fMRT werden die spontanen Aktivitäten im Gehirn gemessen. Diese verlaufen entlang von Bahnen, welche verschiedene Hirnregionen funktionell verknüpfen. Dabei entstehen Netzwerke, („resting state networks“) welche die funktionellen Zusammenhänge verschiedener Regionen aufzeigen, bei denen die neuronale Aktivität zeitlich korreliert ist (Abb. 1).

Dies wird in der Tinnitusforschung häufig angewandt, da davon ausgegangen wird, dass die Tinnituswahrnehmung und die davon ausgehende Beteiligung verschiedener Regionen auch in Ruhe vorhanden ist [14]. Bisher konnten so u. a. 2 nichtauditorische Regionen aufgedeckt werden, welche bei der Chronifizierung, Stressbelastung und kognitiver Dysfunktion bei Tinnitus eine Rolle spielen [24]:

#### Veränderungen im limbischen System

Das limbische System regelt die Verarbeitung von Emotionen und enthält wiederum eine Vielzahl eigener Hirnregionen. Dabei zeigte sich bei TinnituspatientInnen eine ausgeprägtere Verbindung zum auditorischen Kortex und verschiedenen Regionen im limbischen System [26]. Weitere Studien zeigten eine Korrelation zwischen der subjektiven Tinnitusbelastung und der stärkeren Verbindung zwischen auditorischem Kortex und eher limbischen Regionen [5, 12]. Abschließend geklärt ist diese Beziehung noch nicht. Es wird davon ausgegangen, dass das limbische System und die Verbindung bei der Unterdrückung des Tinnitus eine wichtige Rolle spielt und eine Dysfunktion zur Chronifizierung des Tinnitus beiträgt [30].

#### Veränderungen im Aufmerksamkeitssystem

Bei TinnituspatientInnen fanden sich Veränderungen in der Resting-State-fMRT in Regionen und in Verbindungen, welche die Aufmerksamkeit steuern. Eine Untersuchung zeigte eine erhöhte Vernetzung zwischen dem dorsalen medialen präfrontalen Kortex, welcher eine Schlüsselfunktion in Aufmerksamkeitsprozessen hat, und dem auditorischen Kortex. Die entstandenen Korrelationen lassen vermuten, dass dieses Netzwerk in die Wahrnehmung des chronischen Tinnitus eingreifen kann [19].

### Funktionelle magnetische Resonanzspektroskopie

Die funktionelle Magnetresonanzspektroskopie des Gehirns (fMRS) nutzt die MRT, um den Stoffwechsel im Gehirn zu untersuchen. Anstelle einer Aktivität, wie bei der funktionellen MRT, kann die Konzentration von bestimmten Metaboliten in vorher definierten Bereichen (ROI, „region of interest“) des Gehirns gemessen werden. Es wird zunehmend als wichtiges Werkzeug in den Neurowissenschaften etabliert, um die Hirnfunktion unter normalen und pathologischen Zuständen zu untersuchen. Bei Tinnitus konnten auf Basis von Tierversuchen schon verschiedene Metaboliten aufgezeigt werden, welche eine veränderte Konzentration in bestimmten Hirnarealen aufweisen [3]. Dank der fMRS ist eine solche Analyse auch bei Menschen in vivo möglich.

Neuronale Netze verändern sich mit der Tinnituswahrnehmung

**Figure 2:**
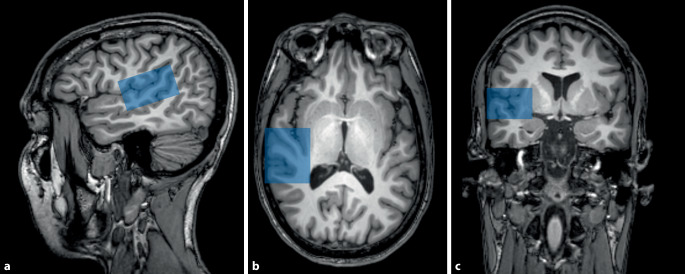

Während Tinnitus weitestgehend im Ohr beginnt, sind auch Elemente der zentralen Hörbahn im Hirnstamm sowie der Hörrinde im Gehirn (auditorischer Kortex; Abb. 2) an der Aufrechterhaltung des Tinnitus beteiligt. Diese neuronalen Netze verändern sich mit der Tinnituswahrnehmung und beeinflussen die Zusammensetzung von Stoffwechselprodukten und Zellen in bestimmten Gebieten des Gehirns. Es gibt Hinweise darauf, dass die Tinnituswahrnehmung auch durch eine fehlende Inhibition (Unterdrückung) von Teilen dieser Netze aufrechterhalten wird [35]. Verdächtigt wird etwa ein Mangel an dem inhibitorischen Neurotransmitter Gamma-Aminobuttersäure (GABA) im auditorischen Kortex oder eine erhöhte Konzentration von exzitatorisch wirkendem Glutamat [15, 36]. In verschiedenen Tierstudien wurden bereits Unterschiede zwischen Konzentrationen solcher Neurotransmitter im auditorischen Kortex aufgezeigt [41]. In der Anwendung von fMRS bei Tinnitus zeigte sich eine reduzierte Konzentration des inhibitorisch wirkenden Neurotransmitters GABA im auditorischen Kortex, was eine erhöhte Spontanaktivität und somit eine weitere Grundlage in der Wahrnehmung von Tinnitus erklären könnte [36].

Die funktionelle MRT bildet aufgrund ihrer sehr guten räumlichen Auflösung den Goldstandard der funktionellen Bildgebung. Bei dieser Technik sind aber 2 bei der Tinnitusforschung wichtige Einschränkungen zu beachten. Zum einen wird ein starkes Magnetfeld erzeugt, welches mit Implantaten im Körper reagieren kann. TinnituspatientInnen mit Cochleaimplantaten, z. B., scheiden somit für Untersuchungen mit dieser Modalität aus. Zweitens erzeugt die MRT laute unregelmäßige Geräusche, welche gerade in Tinnitusfragestellungen eine Unterscheidung zwischen Geräuschwahrnehmung und Tinnituswahrnehmung erschwert.

### Positronenemissionstomographie

**Figure 3:**
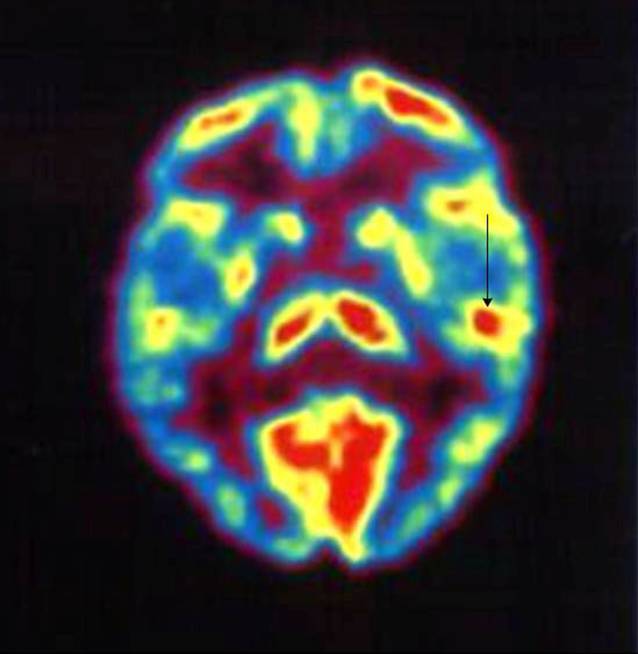

Die PET basiert auf der i.v.-Verabreichung radioaktiver Moleküle, welche am Stoffwechsel beteiligt sind. Durch Nachweis eines hohen Glukoseverbrauchs oder durch einen erhöhten Blutfluss lässt sich ein Rückschluss auf erhöhte Hirnaktivität ziehen (Abb. 3).

In der Tinnitusforschung zeigte sich die PET in der Untersuchung der Hirnaktivität als dienlich. Es wird davon ausgegangen, dass eine periphere Deafferenzierung (z. B. durch periphere Hörschädigung) den Tinnitus auslöst, aber zentrale neuroplastische Prozesse die Wahrnehmung des Geräuschs aufrechterhalten [32]. Zu diesen Prozessen zählen u. a. eine neuronale Hyperaktivität und erhöhte Spontanaktivität im auditorischen Kortex, welche in verschiedenen Tinnitusmodellen Teile der Pathophysiologie sind.

Untersuchungen, bei denen die FDG-PET im Ruhezustand zum Vergleich der relativen Stoffwechselaktivität im linken und rechten primären auditorischen Kortex eingesetzt wurde, ergaben eine signifikant stärkere Asymmetrie bei TinnituspatientInnen als bei Kontrollpersonen, wobei der linke auditorische Kortex eine höhere Ruheaktivität aufwies als der rechte auditorische Kortex [2]. Jedoch wurde in einer neueren Studie keine erhöhte Aktivität in der Tinnitusgruppe festgestellt [9].

Tinnitus tritt signifikant häufiger bei Männern als bei Frauen auf

Da Tinnitus signifikant häufiger bei Männern als bei Frauen auftritt, wurden mittels PET neurobiologische Unterschiede zwischen Geschlechtern getestet. Es zeigte sich, dass Tinnituspatientinnen signifikant höhere Aktivität in den temporalen und parietalen Hirnbereichen aufzeigten als männliche Patienten [8].

Einige Einschränkungen bestehen zur Anwendung von PET: Aufgrund der Verwendung von radioaktiven Molekülen sowie Röntgenstrahlung kann die Untersuchung nicht beliebig oft wiederholt werden. Außerdem ist die zeitliche Auflösung abhängig von der Halbwertszeit des Zerfalls des verwendeten Moleküls. Bei radioaktiv markiertem Wasser entspricht die Untersuchungszeit etwa 2 min. Somit eignet sich diese Untersuchung v. a. dazu, um rasche Veränderungen der neuronalen Aktivität aufzuzeigen, wie etwa nach einer auditorischen Aufgabe. Ein Vorteil liegt v. a. darin, dass die Untersuchung im Vergleich zur MRT sehr ruhig ist. Darüber hinaus können auch PatientInnen mit Implantaten untersucht werden [23].

### MEG/EEG

MEG und EEG zeichnen elektromagnetische Felder des Gehirns auf. Die neuronale Aktivität des Gehirns erzeugt schwache Ströme bzw. Magnetfelder. Neuronen arbeiten immer in Netzwerken und Gruppen, wobei jeweils einzelne Netzwerke für bestimmte Aufgaben zuständig sind (z. B. Verarbeitung von auditorischen Reizen).

Die Verarbeitung von Informationen betrifft somit immer sehr spezialisierte Netzwerke, in welchen es während der Verarbeitung zu synchronen Entladungen kommt. Diese Entladungen von einer Gruppe von Neuronen erzeugt Ströme, welche an der Kopfoberfläche von Elektroden abgeleitet werden. Diese Aufzeichnung von Spannungsunterschieden an verschiedenen Stellen der Kopfoberfläche ermöglicht eine hohe zeitliche Auflösung im Millisekundenbereich. Die räumliche Auflösung ist hingegen begrenzt und abhängig von der Anzahl der Messpunkte (Elektroden) [31].

Die Verarbeitung von Informationen betrifft sehr spezialisierte Netzwerke

Die EEG erfasst Potenzialdifferenzen von elektrischen Feldern, welche an 2 auseinanderliegenden Elektroden abgeleitet werden. Es sind somit immer mindestens 2 Elektroden erforderlich (Referenz- und aktive Elektrode). Die Qualität der Messung und die Genauigkeit der Auflösung hängt also auch mit der Anzahl abgeleiteter Elektroden zusammen.

Bei der MEG wird die Ableitung über ein SQUID („superconducting quantum interference device“) absolut gemessen. Die Messung beruht auf den gleichen Mechanismen wie bei der EEG, wobei nicht die elektrischen Ströme, sondern die daraus entstehenden Magnetfeldschwankungen aufgezeichnet werden. Diese werden durch das umgebende Gewebe wie den Schädelknochen usw. weniger stark abgeschirmt als die elektrischen Signale. Auch sind keine Referenzelektroden nötig. Die Ableitung ist darum räumlich etwas genauer.

Aufgrund ihrer hohen zeitlichen Auflösung sind diese Untersuchungsmodalitäten für die Tinnitusforschung unersetzlich, da sie Informationen zu Veränderungen in neuronaler Verschaltung und Synchronizität bei Tinnituserleben geben können, was bei den Verfahren wie MRT und PET nicht möglich ist. Weitere Vorteile sind, dass die Untersuchung v. a. bei der EEG preiswert sowie nichtinvasiv und nicht radioaktiv sind.

#### Elektro- und magnetenzephalographische Korrelate

**Figure 4:**
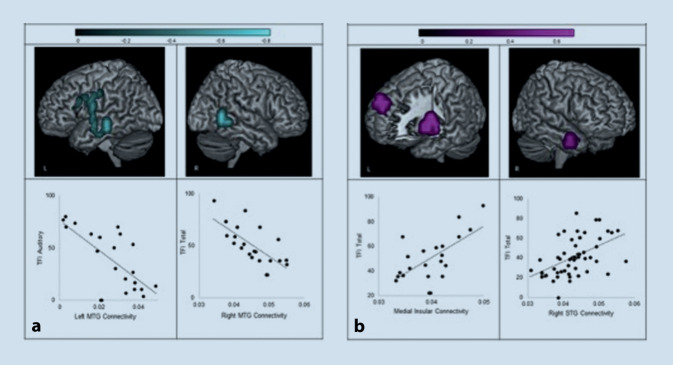

Verschiedene Studien untersuchten Veränderungen der Gehirnwellen bei TinnituspatientInnen. So fanden sich Veränderungen der Spontanaktivität in verschiedenen Hirnbereichen bei TinnituspatientInnen (Abb. 4): Normale Gehirnfunktionen basieren auf einem Gleichgewicht zwischen Inhibition und Exzitation. Je nach situativer Beanspruchung kann dieses Gleichgewicht zugunsten der einen oder anderen Seite verschoben werden. Beispielsweise werden langsame Oszillationen bis 4 Hz Delta-Wellen genannt und treten im Tiefschlaf auf. Alpha-Wellen (8–12 Hz) sind im wachen Zustand bei Entspannung anzutreffen und organisieren die Wahrnehmung von Reizen durch Inhibition. In sensorischen Arealen werden in einem ausgeglichenen Zustand v. a. Alpha-Wellen abgeleitet. Sobald eine erhöhte Anforderung an dieses Areal gestellt wird, z. B. bei aktivem Zuhören, reduziert sich die Alpha-Aktivität in der aktivierten Hirnregion [38]. Es wird vermutet, dass bei Gesunden, diese Alpha-Aktivitätsreduktion nur vorübergehend stattfindet, während bei TinnituspatientInnen eine dauerhafte Reduktion der Alpha-Aktivität ein Hinweis für andauernde auditorische Aufmerksamkeit sein könnte [34, 42].

Eine reduzierte Alpha-Aktivität im auditorischen Kortex wurde in verschiedenen Tinnitusstudien beobachtet: So finden sich in auditorischen Regionen bei TinnituspatientInnen im Vergleich zu einer gesunden Kontrollgruppe verstärkte Delta- und reduzierte Alpha-Aktivität [34, 43]. Interpretiert wird diese Eigenschaft auch im Zusammenhang mit der als Auslöser vermuteten Deafferenzierung nach peripherem Hörverlust, welche konsekutiv zur Reorganisation in den auditorischen Hirnbereichen führt.

Eine reduzierte Alpha-Aktivität im auditorischen Kortex wurde in Tinnitusstudien beobachtet

Basierend auch auf anderen MEG-/EEG-Studien entstand die Theorie der „thalamokortikalen Dysrhythmie“: Unter normalen Umständen beschleunigen die Wellen von einem langsameren Alpha-Rhythmus zu einem schnelleren Gamma-Rhythmus durch die Wahrnehmung von einem (auditorischen) Stimulus. Im deafferenzierten Zustand, wie z. B. nach peripherem Hörverlust, konnte man aber feststellen, dass ein Stimulus die Wellen eher verlangsamt und Delta- sowie Theta-Aktivitäten ableitbar sind. In den nicht vom Stimulus betroffenen angrenzenden Gebieten kommt es aber dadurch zu einer verminderten Inhibition und einer Erhöhung der Gamma-Aktivitäten, was als Ursprung der Tinnituswahrnehmung vermutet wurde [25, 40]. Diese Theorie konnte aber nicht ausreichend bestätigt werden, wobei sich z. T. auch widersprechende Ergebnisse zeigten. Sedley et al. [37] vermuten, dass erhöhte Gamma-Aktivität nicht Ursache, sondern ein Versuch des Gehirns ist, die Tinnituswahrnehmung zu unterdrücken. Aktuell geht man davon aus, dass die verstärkte Gamma-Aktivität für die Tinnituslautstärke verantwortlich ist und nicht für den Tinnitus per se [39].

## Aussicht

Bisher ist die einzige Konstante der mittlerweile zahlreichen Forschungsergebnisse ihre große Heterogenität. Dies ist auch der hohen Variation der Tinnitussymptomatik geschuldet, welche sich nach Ursache, Lautstärke, Tonlage, Dauer, Lateralität, Belastung sowie gleichzeitigem Vorhandensein von Hörminderung oder Hyperakusis unterscheiden kann. Zudem kommt erschwerend hinzu, dass sich alle diese Eigenschaften jeweils nur subjektiv erfragen lassen.

Bis heute fehlt ein objektiver Weg, Tinnitus sicher festzustellen [16]. Die Entdeckung eines Biomarkers, welcher eine Objektivierung des Tinnitusleidens ermöglichen würde, ist mitunter ein aktuelles Ziel der Forschungsbestrebungen. Anforderungen an diesen Parameter beinhalten auch eine Korrelation zur Tinnitusintensität sowie Hinweise auf die Ätiologie, aber auch zur Prävention und zukünftigen Entwicklung einer Therapie.

Aufgrund der großen Heterogenität der TinnituspatientInnen müssen diese Parameter systematisch mit standardisierten Fragebögen erfasst und in großen Datenbanken abgelegt werden, um mögliche Subtypen besser bestimmen zu können [27]. Außerdem kann auch die Bildgebung helfen, in Zukunft Subtypen von Tinnitus aufzudecken. Für die Planung zukünftiger Studien in diesem Bereich gilt es zu fordern, dass die eingeschlossene Patientenpopulation möglichst homogen sein sollte und auch der Vergleich zu einer gesunden Kontrollpopulation (im Idealfall mit vorhandenem und fehlendem begleitendem Hörverlust) als unabdingbare Voraussetzung erfüllt sein muss. Zur weiteren Qualitätssteigerung der von den Autoren vorgestellten Techniken werden zudem Replikationen und Standardisierungen bereits vorhandener Methoden erforderlich sein.

Die funktionelle Bildgebung kann neurophysiologische/anatomische Korrelate von Tinnitus aufzeigen

Die funktionelle Bildgebung kann neurophysiologische/anatomische Korrelate von Tinnitus aufzeigen. Aufgrund der noch eingeschränkten zeitlichen und räumlichen Auflösung der jeweils vorhandenen Bildgebungsmodalität könnten einige bei Tinnitus vorhandenen Veränderungen im ZNS zu klein sein, um entdeckt zu werden. Die Kombination von verschiedenen Modalitäten bei den gleichen PatientInnen kann in Zukunft helfen, die jeweiligen Einschränkungen einer Bildgebung (z. B. mangelnde räumliche Auflösung wie bei der EEG) mit einer anderen Bildgebung (z. B. mit eingeschränkter zeitlicher Auflösung wie bei der fMRT) zu kompensieren.

Aufgrund seines kostengünstigen Einsatzes scheint die EEG das größte Potenzial zu haben, in Zukunft auch häufiger in der klinischen Routine bei TinnituspatientInnen zum Einsatz zu kommen. Elektrophysiologische Korrelate werden bereits experimentell zur Steuerung transkranieller elektrischer und magnetischer Stimulationsverfahren und neurofeedbackbasierter Behandlungen verwendet. Die Erkenntnisse zu veränderter Gehirnaktivität bei TinnituspatientInnen führten bereits zu Therapieansätzen mit Neurofeedback. Dabei wird mittels repetitiven Trainings versucht, die Aktivität in verschiedenen Bereichen des Gehirns bewusst zu steuern. In Versuchen mit der EEG konnte so die Tinnituswahrnehmung mit Hochregulation der Alpha-Aktivität moduliert werden [6]. Alternativ werden auch Neurofeedbackversuche mit Echtzeitmessungen in der fMRT durchgeführt, wobei die Aktivität im auditorischen Kortex verringert werden konnte [10, 21]. Die Fortschritte in der Bildgebung werden weiterhelfen, zuverlässige Modelle der Tinnitusentstehung und -erhaltung zu erstellen, neuartige Therapieansätze zu definieren und in Zukunft auch Therapieerfolge in objektiver Form zu messen [13].

## Fazit für die Praxis

- In Zukunft könnten die elektrophysiologischen Korrelate der auditiven Wahrnehmung dazu dienen, Bevölkerungsgruppen zu identifizieren, die anfällig für die Entwicklung von Tinnitus sind.
- Das wäre ein wichtiger Schritt, um präventivmedizinisch die Belastung des Gesundheitssystems durch viele Tinnitusbetroffene zu verringern.
- Noch ist der Einsatz der funktionellen Bildgebung auf die Anwendung in der Forschung beschränkt.
- Angesichts der Erkenntnisse in der funktionellen Bildgebung sind die Autoren zuversichtlich, dass in Zukunft objektive Marker für verschiedene Tinnitussubtypen identifiziert werden können, beispielsweise durch den Einsatz der EEG.
- Diese Marker könnten dann auch zu der Entwicklung neuer Therapieansätze beitragen.
- Es bleibt allerdings abzuwarten, ob in der Zukunft eine spezifische Bildgebungsmethode einen objektiven Nachweis von Tinnitus ermöglichen wird.
